# A qualitative analysis of relatives’, health professionals’ and service users’ views on the involvement in care of relatives in Bipolar Disorder

**DOI:** 10.1186/s12888-015-0611-x

**Published:** 2015-09-24

**Authors:** Gerasimos Chatzidamianos, Fiona Lobban, Steven Jones

**Affiliations:** 1 Doctorate in Clinical Psychology, Division of Health Research, Faculty of Health and Medicine, Lancaster University, C38, Furness Building, Lancaster, LA1 4YG UK; 2 Spectrum Centre for Mental Health Research, Division of Health Research, Faculty of Health and Medicine, Lancaster University, Lancaster, LA1 4YG UK

**Keywords:** Bipolar disorder, Carers, Mental health, Relatives, Relative involvement, Barrier, Qualitative, Framework analysis, Policy making

## Abstract

**Background:**

Relatives of people with bipolar disorder report that services do not meet their own needs, despite clinical recommendations for the development of care plans for relatives, provision of information regarding their statutory entitlements, and formal involvement in decision making meetings. Further, there is now conclusive evidence highlighting the benefits of relatives’ involvement in improving outcomes for service users, relatives, and the health system as a whole. This qualitative study explored the views of relatives of people with bipolar disorder, service users and healthcare professionals regarding the barriers and the facilitators to relatives’ involvement in care.

**Methods:**

Thirty five people were interviewed (12 relatives, 11 service users and 12 healthcare professionals). Audio recordings were transcribed verbatim and common themes in participants’ narratives emerged using framework analysis.

**Results:**

Participants’ accounts confirmed the existence of opportunities for relatives to be involved. These, however, were limited and not always accessible. There were three factors identified that influenced accessibility namely: pre-existing worldviews, the quality of relationships and of communication between those involved, and specific structural impediments.

**Discussion:**

These themes are understood as intertwined and dependent on one another. People’s thoughts, beliefs, attitudes, cultural identifications and worldviews often underlie the ways by which they communicate and the quality of their relationship. These, however, need to be conceptualised within operational frameworks and policy agendas in health settings that often limit bipolar relatives’ accessibility to opportunities for being more formally involved.

**Conclusions:**

Involving relatives leads to clear benefits for relatives, service users, healthcare professionals, and the health system as a whole. Successful involvement of relatives, however, depends on a complex network of processes and interactions among all those involved and requires strategic planning from policy makers, operational plans and allocation of resources.

## Background

Characterised by recurring mood swings between depression and mania or hypomania [1], Bipolar disorder (BD) is the fourth-leading cause of disability in the world [2]. More than 20 years ago, in his description of an integrated treatment framework, Moltz [3] described the effects of BD on relatives and the wider family. Later studies confirmed some of Moltz’s observations by providing evidence that BD relatives experienced burden at similar levels to those of relatives of people with schizophrenia [4–7] and that this burden correlated with increased physical and psychiatric symptoms in relatives [8].

With the change to care in the community, healthcare provision became more reliant on relatives [9] and put relatives centre stage in government policies internationally (World Psychiatric Association: [10], Canada: [11], U.K.: [12–19], USA: [20], Australia and New Zealand: [21, 22]). BD relatives’ input, in particular, was acknowledged in both the UK National Institute for Health and Clinical Excellence (NICE) guideline [23] and the American Psychological Association (APA) [24] guidelines for BD. There is now evidence for positive outcomes of family-focused approaches and multifamily groups in BD (cf. [25]) that indicated improvements in: recovery from depression and depression symptom severity [26]; recovery from depression, duration of BD episodes, and severity of manic symptoms [27]; relatives’ depression and health risk behaviours and service users’ (SUs) depression symptoms [28]; SUs’ manic or hypomanic relapses [29]; recognition of early warning signs [30], relapse rates [31] and stability in daily routines and emotional self-regulation strategies [30]. Further, narratives from qualitative interviews suggested that involving relatives is perceived as valuable in relapse prevention [32].

In the UK, the ways that relatives can be involved has been described in the Triangle of Care report [33, 34]. Specifically, the report emphasised a strategic involvement of relatives that spans across planning and provision of healthcare, which is not restricted only in settings that offer structured family interventions (e.g. Family Focused Treatment [35]). Further, clinical guidelines for BD suggest that relatives’ own needs should be identified via individualised assessments that form the basis for the most suitable form of involvement [23]. These assessments should involve care plans for relatives and provision of information and advice regarding relatives’ statutory rights. The assessments should also provide the space and time for advanced statements to be developed and for planning decision making meetings attended by all relevant parties including relatives. Despite this evidence, the latest systematic review on the burden experienced by BD relatives highlighted the “need to better understand caregivers’ views and personal perceptions of the stresses and demands arising from caring for someone with BD in order to develop practical appropriate interventions and to improve the training of caregivers” [36].

Understanding the barriers and facilitators (B&Fs) to relatives’ involvement is, therefore, very important as it can potentially help address the challenges being identified and inform strategic planning that would support relatives in being effectively involved. This is particularly important for relatives of people with BD for various reasons. The literature in relatives’ involvement has focused primarily to people with psychosis [37, 38]. This might not be sufficient to account for BD relatives’ increased use of mental health services for their own challenges with depression and anxiety due to the increased burdens experienced [4]. Importantly, there is evidence suggesting that the exact fluctuating nature of mood swings, which is associated with BD, often leads to increased levels of anxiety for relatives, who worry about a potential forthcoming mood episode even when SUs’ mood appears relatively stable [8]. Further, the structure of services is such that SUs engagement is restricted only when their mood is elated or depressed. Rapid changes that could escalate to suicidal or self-harming behaviour might occur before services can be involved, which leaves relatives as the only available source of support. It is also established that services are designed to respond better to chronic rather than episodic presentations of problems, with healthcare professionals feeling more equipped to manage schizophrenia as opposed to BD [39]. Also, with people with BD being more likely of having children or being involved in a married/cohabiting relationships compared with psychosis [40], intrafamilial relationships are more at risk of being strained.

Consequently, the generalisation of findings form the literature on psychosis might be misleading due to BD specific characteristics that present unique challenges in the effective and meaningful involvement of relatives. Hence, complementing the work previously done, this paper reports B&Fs in relatives’ involvement from the perspectives of relatives, healthcare professionals (HPs) and SUs. The research question was: What are the barriers and the facilitators to relatives’ involvement in BD?

## Methods

### Ethics statement

All research methods and procedures complied with the Helsinki Declaration as revised in 2013 (64^th^ World Medical Association General Assembly, Fortaleza, Brazil), were sponsored by Lancaster University and had received favourable opinion by the Yorkshire and the Humber National Health System (NHS) Research Ethics Committee (REC) (REC number: 11/YH/0174) and the R&D of Cumbria Partnership NHS Foundation Trust (study reference number: 3011).

### Design

All interviews were conducted individually and in private having obtained written consent individually, and direct quotations from the interview’s transcripts are reported anonymously. Participants’ responses were triangulated and analysed using framework analysis [41], which has been described as “an excellent tool to assess policies and procedures from the very people that they affect” [42]. The focus was on individual perspectives rather than shared experiences of a particular service, so there was no requirement that SU, relative and HP participants were specifically linked. In keeping with the public involvement in research agenda, the design and conduct of the study was developed in consultation with a SU researcher with a diagnosis of BD, a relative of a person with BD and a relative of a person with psychosis. It is believed that this enhanced both the quality and the relevance of the research, and enabled us to conduct the project ‘with’ the members of the public rather than ‘to’, ‘about’ or ‘for’ them. Their involvement ensured that the project is in accordance with broader democratic principles such as citizenship, accountability and transparency [43]. Finally, the reporting of this manuscript adheres to the Relevance Appropriateness Transparency Soundness guidelines (RATS) [44].

### Participants

Thirty five self-referred volunteers took part in the study (11 SUs; 12 relatives; 12 HPs). Relatives had to be in regular contact with a BD service user (minimum 3 contacts/week) and have an emotional and/or practical supporting role in the service user's life (i.e. be in a (i) family, (ii) friend, or (iii) partnership relationship with a service user); aged over 18 years.; and able to understand spoken and written English in order to provide informed written consent and participate in interviews. In contrast to the term ‘carer’, ‘relative’ acknowledges the multiple facets of the relationship between the one who provides and the one who receives care. This was the preferred term of the relatives and SUs who were involved in the design of this study. We therefore use ‘relative’ to refer to a parent, partner, child, sibling, friend, neighbour or an extended family member who offers unpaid support to a person with BD. SUs had to be aged between 18 and 65 years., meeting a research BD diagnosis (I, II or NOS) established by the SCID DSM-IV interview [45, 46]. SUs also had to be able to understand spoken and written English in order to provide informed written consent and participate in interviews (in line with the procedures outlined by Nicholson, Cutter and Hotopf [47]). HPs had to be employed by the NHS in the UK or other non-statutory services which serve people with BD and relatives, and to have had experience working with this population. HPs from any professional background could participate. Participants self-referred into the study having responded to recruitment campaigns in electronic and print media and advertising. Only those who did not meet inclusion criteria were excluded from the study. This was decided by the first author, who was responsible for responding to potential participants’ enquiries and recruitment. At the time of participation in this study, neither SUs nor relatives received formal family therapy. Key characteristics of the sample appear in Tables 1 and 2.

**Table 1:** Summary of clinical and demographic characteristics of service users’ interviews

	n%
Age - mean(σ_X_)	47.36 (13.40)
Diagnosis	
BD I	9 (82)
BD II	1 (9.09)
Schizoaffective Disorder (Bipolar)	1 (9.09)
Age at first mood disorder diagnosis - mean(σ_X_)	27.18 (8.68)
Age at diagnosis of bipolar disorder - mean(σ_X_)	34.91 (12.05)
Number of previous episodes of depression	
1-6	5 (45.45)
7-11	1 (9.09)
12-29	1 (9.09)
≥30	4 (36.36)
Number of previous episodes of hypo/mania	
1-6	7 (63.64)
7-11	2 (18.18)
12-29	1 (9.09)
≥30	1 (9.09)
Number of previous hospitalisation	
0	2 (18.18)
1-6	8 (72.73)
7-11	1 (9.09)
Highest level of education	
Secondary	1 (9.09)
Further	5 (45.45)
Higher	5 (45.45)
Employment status	
P/T	2 (18.18)
Retired	2 (18.18)
Voluntary	2 (18.18)
Unemployed	5 (45.45)
Gender	
Female	6 (54.55)
Male	5 (45.45)
Ethnic origin	
White British	11 (100)
Marital Status	
Single	6 (54.55)
Married	3 (27.27)
Divorced	2 (18.18)
Living arrangements	
Partner only	2 (18.18)
Alone	7 (63.64)
Children only	1 (9.09)
Parent/s only	1 (9.09)
Indices of Deprivation by postcode 2010*	
lower quartile (least deprived)	3 (27.27)
mid low quartile	2 (18.18)
median	1 (9.09)
mid upper quartile	2 (18.18)
upper quartile (most deprived)	3 (27.27)
IQR**	28.248
Religion or belief	
Buddhism	1 (9.09)
Christianity	5 (45.45)
None	3 (27.27)
Agnostic	2 (18.18)

*Postcodes were converted to Lower Layer Super Output Areas and categorised into quartiles in keeping with the English Indices of Deprivation 2010 [71]

**IQR = Q_3_ − Q_1_

**Table 2:** Summary of clinical and demographic characteristics of relatives and HPs

	*n*%	
	Relatives	HPs
Age - mean(σ_X_)	60.08 (8.43)	43.17 (8.82)
Highest level of education		
Secondary	4 (33.33)	0
Further	4 (33.33)	0
Higher	4 (33.33)	12 (100)
Employment status		
FT	2 (16.67)	8 (66.67)
P/T	4 (33.33)	4 (33.33)
Retired	6 (50)	0
Gender		
Female	7 (58.33)	7 (58.33)
Male	5 (41.67)	5 (41.67)
Ethnic origin		
White British	11 (91.67)	11 (91.67)
White any other	1 (8.33)	0
Indian	0	1 (8.33)
Marital Status		
Single	1 (8.33)	1 (8.33)
Married	10 (83.33)	7 (58.33)
Cohabiting	1 (8.33)	1 (8.33)
Separated	0	1 (8.33)
Living arrangements		
Partner only	9 (75)	5 (41.67)
Partner plus children	1 (8.33)	5 (41.67)
Alone	2 (16.67)	1 (8.33)
Children only	0	1 (8.33)
Indices of Deprivation by postcode 2010*		
lower quartile (least deprived)	3 (25)	3 (25)
mid low quartile	3 (25)	3 (25)
mid upper quartile	4 (33.33)	3 (25)
upper quartile (most deprived)	2 (16.67)	3 (25)
IQR**	3.41	21.5
Religion or belief		
Atheism	1 (8.33)	6 (50)
Buddhism	0	1 (8.33)
Christianity	8 (66.67)	3 (25)
Church of England	1 (8.33)	1 (8.33)
Hinduism	0	1 (8.33)
None	2 (16.67)	0

*Postcodes were converted to Lower Layer Super Output Areas and categorised into quartiles in keeping with the English Indices of Deprivation 2010 [71]

** IQR = Q_3_ − Q_1_

### Procedure

The first author conducted in-depth semi-structured interviews of 45–60 min that took place at Lancaster University or other sites chosen by participants (e.g. homes, NHS sites, community places). On the day of the interview, the interviewer reiterated the Participant Information Sheet to the participants, explained the limits to confidentiality, and obtained written consent. Participants then completed a demographics questionnaire. The topic guide used (available on request) helped with focussing the interview around (i) participants’ definitions of relative’s involvement in the mental healthcare team, (ii) participant perspectives on the value of involving relatives, (iii) examples of barriers and facilitators to involving relatives in mental healthcare of people with BD, and (iv) the required support if relatives were to be involved. Open ended questions were used to help the researcher better understand participants’ experiences of relatives’ involvement as opposed to seeking answers to direct questions. Interviews were audiotaped and were later transcribed verbatim by a professional transcriber, who had previously signed a confidentiality agremeent.

### Analysis

The 5 key stages of framework analysis were followed: familiarization; identifying a thematic framework; indexing; charting; and mapping and interpretation [41]. Framework analysis is a common method used in health research as it helps with organising and managing qualitative data through a process of summarisation that results in a matrix output [48]. This provides a structure where analysts can systematically reduce the data by analysing them by participant and by coding. The first author of this paper conducted the interviews, read the transcripts, listened to the audiotapes, identified key ideas and recurrent emerging themes and developed the preliminary thematic framework. These were discussed with members of the research team in an iterative process, which resulted in the final consensus framework. Interview extracts were then tabulated and interpreted taking into consideration the existing literature. ATLAS.ti was used to aid analysis. This is a platform that assists with the uncovering and systematically analysing complex phenomena hidden in unstructured data commonly used in qualitative research (e.g. text) [49]. The research team had varied experience in clinical and research areas including family interventions; the first author is a clinically trained experimental psychologist specialised in family interventions and also a university lecturer, and both co-authors are senior clinical academics (Professors of Clinical Psychology). None of the authors occupied dual roles (clinical and researcher) with any of the participants. This diversity has enriched the conceptualisation of the framework product.

## Results

**Fig. 1:**
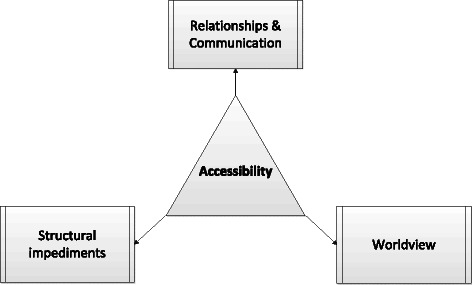
Map of theme and sub-themes of B&Fs to relatives’ involvement

This study set out to identify the B&Fs to relatives’ involvement in the treatment of BD as experienced by relatives, HPs and SUs. Overall, narratives in this study confirmed difficulties in involving relatives described in the literature for both BD [50] and psychosis [51]. Participants recognised that there are opportunities for support for relatives to be involved, which are in keeping with existing clinical guidelines [23] and some evidence [32]. The opportunities to be involved, however, appeared limited and not easily accessible. As such ‘Accessibility’ was identified as the main theme that captures the B&Fs to relatives’ involvement. Accessing the available sources of support, we found, was influenced by three main factors. These were i. pre-existing worldviews, ii. the quality of relationships and communication, and iii. other structural impediments. These are perceived as intertwined and dependent on one another (Fig. 1).

### Pre-existing worldviews

This theme emerged from narratives attributing relatives’ exclusion to pre-existing worldviews of healthcare provision and specific negative attitudes towards involvement.

I think a lot of it [relatives’ exclusion] is from the attitudes by social services and by medical people about following agendas and not wanting someone tricking them up and change the direction of things (Relative).

I think this is a cultural thing, it is very difficult to accept, but some doctors don’t like to be told [what to do] (SU).

Relatives’ involvement is often perceived as not carrying any relevance to practice, as in the case of HPs who construct causal relationships between relatives’ actions and SUs’ challenges.

[HPs] often have a formulation of the person’s difficulties that involves the families and how they treated the person badly (HP).

Similarly, the westernized medically driven emphasis on individual SUs, rather than their social context, leads to the needs of support networks being perceived as secondary, despite the fact that some SUs would welcome support to be provided directly to their relatives even in their absence.

There is a cultural positioning of problems within a person, as a psychiatric medical tradition positioning problems in a person (HP).

I’d rather not be there, I think they [relatives] should have time for themselves because… it’s not about me, is it? [It] is about other people as well (SU).

A participant perceived relatives’ involvement as a fundamental aspect of the relatives’ role with respect to the individual SU.

Relatives’ involvement is natural. It just happens, most of people they just do it naturally, they care for the person who are related to. So when the person’s health state changes, they do something to try to help, to try to rectify the condition. (…) Families generally have a natural response to that; they try to help, (…) so there is nothing you can do really to stop that (HP).

Finally, a SU thought that relatives’ involvement can be facilitated if all parties (relatives, SUs and HPs) are actively engaged in efforts to learn together how best to implement care.

It is some learning exercise between all of us. But I think, it’s when this triangle is made firmly at the beginning, at the very beginning and works on and, and everybody listens and learns. (…) As service users are crucial in this, we’ve got to want to learn, we’ve got to want to get better, we’ve got to learn to manage, if we are unwilling to learn that’s a sadder place to live (SU).

### Quality of relationships and communication

A key component for effective involvement is the presence of trusting collaborative relationships between partners. Negative dynamics appear to constantly impede such relationships; in particular through power struggles between relatives and HPs. There seemed to be a sense of ownership of the SU and an underlying need to convince one another that each party knows the SU the best, which increased the risk of the SU being distanced from any decision process. In effect, even positive intentions from either relatives or HPs might result in SUs losing their autonomy.

There can be a competitive dynamic about who can look after the patient best. Is it the family or is it us? (HP).

[HPs] are the knowledge of mental illness but we [relatives] are the practical side… you know, we are the inside knowledge (Relative).

SUs, however, often objected to the involvement of their relatives in order to protect them from becoming stressed by the whole experience.

When I was in hospital, I requested the care coordinator didn’t contact my mother, because I thought she would stress out (SU).

Poor communication among relatives, SUs and HPs was a common theme in reducing accessibility to available services. The use of technical language by HPs, for instance, coupled with patronising or disempowering language were all described as hindering effective involvement.

There needs to be a clear understanding on the part of HPs, a sensitivity to the kind of the words used. They could be disempowering kind of words, or expert kind of words (…) anything that can be patronizing, especially when the carers are coming from a position of, you know, actually I’ve got the richness of experience of that, because I live with the person (HP).

However, there were also narratives from relatives expressing empathy towards HPs for the difficulties they face in delivering the service they might want to.

We’re talking, talking, talking but we can’t do anything about it and I think they [HPs] find that difficult too because they feel impotent but they can’t do what they want to do, they can’t give a service that they want to give (Relative).

The quality of communication between relatives and SUs also was often found to be influenced by the episodic nature of BD.

At the moment my daughter would be very very happy because we’re involved and when she is in a high state, [she] doesn’t want to know (Relative).

The question mark for me [SU] is whether SUs should have that right to state that they wanted to have their brothers excluded from communications especially when we are non-compos mentis (SU).

Different needs at different phases in peoples’ journeys of caring or experiencing BD warrants different types of support. But narratives here suggest relatives described being listened to and good communication with HPs as the two key areas of support needed.

I only think they [relatives] need the communication with the HPs, I don’t think they need any more support than that (Relative).

Lack of communication with the professionals and the carers is a big, big issue and not being listened to as a carer (Relative).

The structure of services is such that relatives seldom have access to the same HPs. This reduces the likelihood of developing collaborative and trusting relationships, hindering the quality of communication.

The relationship with HPs is beyond fluid. It’s sort of ridiculous sometimes, (…) because they [relatives] want the consistency, they want to be dealing with somebody that they know, that they trust, that get consistent information from (HP).

Collaborative partnerships with relatives are facilitated, however, when HPs are not defensive, are open to criticism, acknowledge service limitations, provide re-assurance and recognize the challenges that relatives experience, and do not communicate in paternalistic ways toward relatives.

You have to be willing not to being defensive and really open to hearing criticism and acknowledging that something is around you, you can’t control in other ways (HP).

We should stop being paternalistic towards relatives by assuming that this information would be too painful (HP).

Effective involvement also occurs when HPs address openly that no-one is to be blamed, accommodate relatives’ anger and disappointment toward services, and give time to grieve.

[HPs can say] It is ok for you to be angry. I understand you have had bad experiences. And then just giving them [relatives] time to ventilate really (HP).

Finally, HPs perceived the limited understanding of confidentiality, and how it is used or abused, to predispose people’s behaviours and attitudes, and that it has direct implications on their communication.

Staff potentially use [confidentiality] as an excuse (HP).

We hide behind the confidentiality thing sometimes because it’s complicated, yeah? If someone wants to talk to you, you’re worried about confidentiality, you then have to end the conversation, you have to go and find the SU, you have to get the SU’s consent (HP).

[HPs] are frightened of confidentiality; they are frightened that the clients will not like it if you try to share information about them with other people, including the carers (HP).

Relatives’ involvement, however, should not be applied uniformly as it might not always be appropriate due to risks of over monitoring or pathologizing.

Carers can excessively monitor, so I have people say, so we’re not allowed to enjoy ourselves, we can’t be happy, we can’t sit home and watch TV and burst on laughing because the family will go, oh what’s wrong? (SU).

### Structural impediments

Real progress requires organizational changes at all levels. Currently, several structural impediments leave HPs with limited opportunities to involve relatives; because of institutionalised resistances to relatives’ involvement and because HPs operate in a work environment in which managers do not always set realistic expectations.

I think there is an institutionalised sort of resistance over other people’s involvement (Relative).

[Managers] force staff to do things when (…) it relates to kind of payment by results, or risk stuff that can show up in a newspaper. But when it comes to family work and ideas about family work, they go, hmmm, oh yeah! It’d be nice if the staff start to use this (…) that’s reckless because what they need to do is just stronger leadership management (HP).

We’ve got too many demands upon people (…). So if I am saying to you, right, you’ve got to do some family therapy. The manager says to you, you’ve got to do your fire lecture. And I need the fire lecture by the weekend. You’ve got to do the family therapy later because you think I’ve got 2 and half years to do this. But of course by next week (…) it would be something else, (…) and it would be a new file that we need you to take. And then send it to me into 2 h. So I think a lot of these pressures of overwhelming numbers of requirements impair people’s willingness and ability to do stuff (HP).

This is further exacerbated by the fact that some practices designed to include relatives do not in practice do so. For instance, although Carer’s Assessments could lead to some positive outcomes (such as carers’ breaks), many participants described them as procedurally irrelevant to relatives’ needs.

I take it [Carer’s Assessment] along and say would [you] like to do the form? And they [relatives] look at it and say that’s got nothing to do with my needs (HP).

Besides, in most services, sessions with a relative are not part of the workload allocation; only time with service users counts. In effect, relatives might not being seen at all, or rather paradoxically, some contacts with relatives might be unaccounted for just because they cannot be recorded.

We get criticized that our contacts are not as high as they should be. But their choice of how they’ve chosen to measure contacts is not in 20 min segments. It’s just a pure contact. So if I phone you up and said I will be coming late for our appointment today, that could count as a contact. Equally, I could spend 3 h with you and all of your family… that’s one contact (HP).

Further, offering services to relatives only during work hours limits relatives’ accessibility to support mechanisms.

Beyond 5 o’clock the whole medical team shuts down (Relative)

It’s not very easy to get an appointment after 5 o’clock either with care coordinator, or to meet a psychiatrist, or to meet a psychologist or during weekend (HP).

In contrast, central recognition that involving relatives is not an optional add-on would allow HPs to prioritize allocating time to relatives.

Some top down recognition that [involving relatives] is part of your job and not an add-on, is very helpful in enabling you to prioritize it (…) whether that is in your supervision or operational approach protocols (HP).

Furthermore, policy makers need to ensure that health agendas and policies are supported by financial drivers to support their implementation.

But if it [Act] is not backed up with cash then it crawls (…). So it’s all inspirational rather than implementational. If all the relatives and carers were thrown out of town, the whole country would be absolutely [profanity] and we would have banking crisis every year (HP).

The system can show commitment to relatives’ involvement by offering HPs better training and clinical supervision, so that they can be re-assured about the service they offer, or be guided when feeling concerned or defensive over their training and skills.

When you’ve been defensive or concerned, or complaints have been expressed, you do need to talk it through with one you feel like they’ve got your back a bit really, as a practitioner. You might not have done anything wrong, but sometimes you have done stuff wrong. You have made a bit of a cock-up and you need to be able to access someone to talk that through (HP).

To conclude with, a participant summarized the B&Fs to relatives’ involvement in the following quotation.

There are 3 things that obstacle (sic) people doing stuff: hill, will and skill. ‘Skill’ is gonna be a part, people’s gonna have basic communication skills, some basic group management skills and basic educational skills and the ability to explain themselves to families (…). ‘Will’ is about, do they believe that it’s of some importance? (…) they would be some in the health service who don’t believe that it’s the parents’ business (…). The other thing is the ‘hill’, which is just the ‘but’. In an organization like the NHS there are far too many barriers that impose in somebody’s abilities, the requirements of the organization as opposed to the requirements of the task (HP).

## Discussion

We explored relatives’, SUs’ and HPs’ understanding of relatives’ involvement in mental healthcare teams of people with BD. We identified that that key barrier to relatives’ inclusive care in BD fell under an overarching theme of ‘accessibility’ to the available opportunities for support. Relatives accessing the available opportunities for collaborative care appears to be influenced by people’s pre-existing worldviews, the quality of relationships and of communication among stakeholders and specific structural impediments. An attempt was made to also explore whether the experiences of relatives vary based on how much contact there is between HPs and relatives. This, however, was not possible for four main reasons. Firstly, there is no measure available that would enable the quantification of the contact between relatives and HPs in a meaningful way. Secondly, the number or mode of contacts between the two parties is so diverse that does not enable group comparisons. Finally, the number of contacts would not necessarily provide an insight as to whether these have resulted in a positive or a negative experience. Finally, the quantification of contacts and its implications to relatives’ experiences of involvement would be more suitable to a quantitative study that was designed to explore that, which was not the focus of this qualitative investigation of people’s experiences.

Participants (HPS, relatives and SUs) reflected on whether the provision of mental healthcare is relevant to relatives, and if so to what degree. Thoughts, beliefs, attitudes, cultural identifications and worldviews, are fused together and either foster or block involvement. Similarly to Kaas et al. [52] and Winefield and Burnett [53], our participants viewed issues such as perceived lack of skills or confidence and negative attitudes as major barriers to developing partnerships with relatives. Such beliefs are often culturally bound. Relatedly, Wong [54] reported on how traditional Chinese values and beliefs informed relatives’ expectations of themselves and of the person with mental health problems and perceived their obligations. Similar barriers were related to people’s beliefs around a culturally influenced debate such as whether the HPs should serve the individual or their relatives. Participants referred to South Asian cultures where relatives’ involvement is often the default position. This is not always a positive involvement, however. Researchers studying South Asian populations suggest that relatives’ involvement could lead to SUs’ isolation or even delayed access to treatment [55]. Nevertheless, the debate is yet again centralized around two notions, which seem to be perceived as competing (i.e. service user vs. relative – individual vs. collective). Successful partnerships require these two to be seen as complementary.

Herrman, et al. [56] described good mental health as the ability to sustain mutually satisfying and enduring relationships. Also reflected in our data, we found that the quality of the relationship and communication between parties often underlies the process by which collaborative partnerships between HPs, SUs and relatives are built. Rowe [57] concluded that professional negative attitudes to communication and disengagement with carers posed detrimental barriers that upheld relatives’ rights to fulfil their caring role, arguing in favour of a more empathic communication by professionals. Similarly, Stiberg et al. [58] found that using a video learning tool increased HPs’ empathy and the clinical competence to initiate and sustain good quality relationship and communication with relatives.

A common stumbling-block in the communication between HPs and relatives was the use/abuse of ‘confidentiality’. This is not the first time that the notion of confidentiality was found to hinder involvement [59]. Specific guidelines, however, have been developed that describe how HPs can share information with relatives without breaking confidentiality or even when service users do not consent for information to be shared [60–63]. Sharing with relatives of people with BD is also supported by the latest NICE guidelines [23]. HPs who are aware of the different categories of information, identified by Pinfold et al. ([64]; i.e. general, personal, personal sensitive), would be in a better position to share appropriate information with relatives without compromising confidentiality. Future research should assess how effective and feasible to implement these guidelines is, and any possible impact on changing the culture of involving relatives.

Lack of formal support from policy makers and poor leadership management seems to block involvement, despite the fact that it is encouraged by NICE [23]. Because of the fact that the issues appear to remain unresolved, exploring the barriers to implementing existing guidelines should be prioritized over developing new ones, especially because the percentage of HPs adhering to mental health guideline could be as low as 27 % [65]. In addition to the challenges to implement guidelines described in Francke et al. [66], a major barrier we identified is the lack of financial drivers and concrete operational plans to support any proposed changes.

Participants also said that relatives’ involvement should be prioritized across all levels of healthcare provision. This could take the form of staff training; the lack of which is what Kim and Salyers [67] attributed HPs’ negative attitudes to. Heru [68] stressed that working with relatives should be emphasized in undergraduate mental health training following a three tier model consisting of knowledge, skills and attitude, allowing students to understand family systems, how these interact with both psychiatric experiences and recovery patterns, and to formulate biopsychosocial interventions. Based on our results, HPs also need to be able to tolerate relatives’ distress so that this does not lead to avoidance. They need to accept the limitations of the services they offer to relatives, and to acknowledge that at least some of relatives’ dissatisfaction stems from the fact that however well the healthcare system might try to respond, it cannot meet the relatives’ expectation of gaining their lives back. The skills needed to perform these tasks could be enhanced through clinical supervision, the limited availability of which appears to be a major barrier to involving relatives. This is consistent with previous accounts that described the barriers to establishing clinical supervision in acute mental health inpatient units [69]. Relatives’ involvement, however, is not a panacea and hence not all relatives should always be involved; instead, HPs need to make a judgment call as to when and how certain relatives can be involved in each individual case.

Finally, as reflected by the quotations reported in this paper, our participants seem to focus on the barriers to relatives’ involvement more than the facilitators, despite the fact that the interviews were designed to elicit both B&Fs in equal parts. Research on ‘negative bias’ could potentially explain our participants’ preference to focus on the negatives (barriers) rather than the positives (facilitators) to the involvement of relatives [70]. However, as negative bias was not the focus of this study, no conclusive arguments can be drawn regarding our participants’ possible tendency towards barriers as opposed to facilitators to relatives’ involvement.

### Limitations

Participants identified several facilitators to involving relatives. We have not tested these as to whether they would improve outcome for all partners in care. We anticipate, however, that this discussion will trigger further research and provide ideas for ways forward to mental health policy makers. Our data derived from 35 interviews with relatives, SUs and HPs. Although this is a large sample size in comparison with other qualitative research in this area, these results should be treated with caution when attempting to generalise across all relatives of people with BD. This is because the data come exclusively from interviews with people who live or work in the North West of England, and therefore the results reflect primarily the British working culture, healthcare organizations, forms of education that are typical of the UK and the socioeconomic makeup of the region. Specifically, only one participant was not White (1 HP). Whilst this reflects the make-up of the population of the North West of England, the findings might not apply to families in the UK from a non-White ethnic background or those which carry a cultural heritage from diverse historical circumstances, or immigration histories. As discussed earlier, however, the consistent international evidence regarding relatives’ experiences suggests that the findings could potentially apply to other countries, which have similar healthcare systems to that of the UK particularly around service access and delivery. Further, as indicated in Tables 1 and 2, participants held diverse religious beliefs and therefore it can be argued that the present findings reflect views that are informed by a variety of religious practices.

Despite the diverse background and experience of the authors, not all sources of bias can be ruled out, as the authors pre-existing understanding of relatives’ involvement in BD would have a priori influenced the development of the topic guide and the results identified. The use of SUs and relatives in the development of the topic guide and the iterative analysis process that allowed the different perspectives of each author to be incorporated in the final framework product, however, should have increased the objectivity of data reporting. One potential limitation of the study, however, is the requirement that relatives had to have a minimum of 3 contacts/week with the SU. This results in relatives with less contact/week to be excluded from the study, and hence the findings of the current study may not generalize to all SUs. This criterion, however, was set mainly to ensure that the relatives included in this study played an instrumental role in supporting the SUs and frequency of contacts might be one of the parameters that could define the volume of support.

## Conclusion

By triangulating the views of relatives of people with BD, SUs, and HPs it appears that opportunities for relatives to be involved are indeed available and these are supported by clinical guidelines. Despite their availability, however, these opportunities are often limited or inaccessible due to pre-existing worldviews of the parties involved, the quality of their relationships and communication and other structural impediments. Our results suggest that involving relatives leads to clear benefits for relatives, SUs, HPs, and the health system as a whole. Successful collaborative partnerships between all parties, however, depends on a complex network of processes and interactions among all those involved and requires strategic planning from policy makers, operational plans and allocation of resources. Clinical research has traditionally focused on the effects of interventions on those carrying a diagnosis. Informed by qualitative accounts such as those reported here and in addition to outcomes for SUs, it would be worth for future research to explore primary outcomes from relatives that derive from relative inclusive service provision. Such data would enable clinicians and policy makers to develop interventions that meets the needs of all those involved in a holistic/systemic manner.

## Abbreviations

BD: Bipolar disorder
SU(s): Service user(s)
HP(s): Health professional(s)
B&Fs: Barriers and facilitators
REC: Research Ethics Committee
R&D: Research and Development
NICE: National Institute for Health and Clinical Excellence
APA: American Psychological Association
